# Evaluation of a large healthy lifestyle program: informing program implementation and scale-up in the prevention of obesity

**DOI:** 10.1186/s13012-016-0521-4

**Published:** 2016-11-24

**Authors:** S. L. Kozica, C. B. Lombard, C. L. Harrison, H. J. Teede

**Affiliations:** 1 Monash Centre for Health Research and Implementation (MCHRI), School of Public Health and Preventive Medicine, Monash University, Locked Bag 29, Monash Medical Centre, Clayton, Victoria 3168 Australia; 2 Endocrinology and Diabetes Unit Monash Health, Clayton, Victoria Australia

**Keywords:** Obesity prevention, Evaluation, Implementation, RE-AIM framework, Rural and program effectiveness

## Abstract

**Background:**

The Healthy Lifestyle Program for women (HeLP-her) is a low-intensity, self-management program which has demonstrated efficacy in preventing excess weight gain in women. However, little is known about the implementation, reach, and sustainability of low-intensity prevention programs in rural settings, where risk for obesity in women is higher than urban settings. We aimed to evaluate a low-intensity healthy lifestyle program delivered to women in a rural setting to inform development of effective community prevention programs.

**Methods:**

A mixed method hybrid implementation and evaluation study, guided by the RE-AIM framework (addressing the Reach, Effectiveness, Adoption, Implementation, and Maintenance), was undertaken. Data collection tools included anthropometric measures, program checklists, questionnaires, and semi-structured interviews with participants and local stakeholders. The RE-AIM self-audit tool was applied to assess evaluation rigor.

**Results:**

Six hundred and forty-nine women from 41 relatively socio-economic disadvantaged communities in Australia participated: mean age 39.6 years (±SD 6.7) and body mass index of 28.8 kg/m^2^ (±SD 6.9). A between-group weight difference of −0.92 kg (95% CI −1.67 to −0.16) showed program effectiveness. Reach was broad across 41 towns with 62% of participants reporting influencing some of the health behaviors of their families. Strong implementation fidelity was achieved with good retention rates at 1 year (76%) and high participant satisfaction (82% of participants willing to recommend this program). Over 300 multi-level community partnerships were established supporting high adoption. Stakeholders reported potential capacity to implement and sustain the prevention program in resource poor rural settings, due to the low-intensity design and minimal resources required.

**Conclusions:**

Our comprehensive RE-AIM evaluation demonstrates that an evidence-based obesity prevention program can be successfully implemented in real-world settings. The program achieved broad reach, effectiveness, and satisfaction at the community and stakeholder level, revealing potential for program sustainability. The evaluation addressed implementation knowledge gaps to support future obesity prevention program scale-up.

**Trial registration:**

Australian and New Zealand Clinical Trial Registry ACTRN 12612000115831 [http://www.anzctr.org.au/].

**Electronic supplementary material:**

The online version of this article (doi:10.1186/s13012-016-0521-4) contains supplementary material, which is available to authorized users.

## Background

Treatment of established obesity via lifestyle interventions is challenging with poor engagement, high costs, and minimal sustainability [1, 2]. Weight loss is difficult to achieve at the individual level due to adaptive physiological responses post-weight loss, which almost universally drives weight regain [2, 3]. At the individual level, weight gain prevention is feasible, requiring only minor modifications to energy intake and expenditure [4]. At the system level, primary prevention programs have the potential to reduce health care costs [1, 5]. In this context and with alarming escalation in obesity rates internationally, the World Health Organization (WHO) has identified prevention of excess weight gain as an international health priority [6].

Government prevention agencies have advocated the need for weight gain prevention programs in high-risk populations. Women have high rates of unhealthy weight gain [7, 8], and in many world regions, women have greater obesity prevalence than men [9] with higher subsequent obesity-related complications [10, 11]. Longitudinal data reveals that 20% of reproductive-aged women within a healthy weight range will become overweight within 5 years [12]. For most individuals, weight gain is gradual over several decades and estimated at 600–800 g per year [4]. Notably, even slight increases in weight of ~0.6 kg per year have been shown to increase the risk of breast cancer [13], hypertension [14], type 2 diabetes [15], and coronary heart disease [16] in women. Furthermore, as reproductive-aged women often have a key role in determining household food choices and sedentary behaviors, the need to invest in strategies to prevent excess weight gain in women is clear. In this context, we developed a low-intensity healthy lifestyle program for women, The Healthy Lifestyle Program for women (HeLP-her), shown to have efficacy for preventing weight gain in women in prior RCT’s in different settings and ethnically diverse populations [4, 17–19].

In developed countries, rural-dwelling women are more vulnerable than urban-dwelling women with lower socio-economic status and elevated rates of weight gain and obesity [20, 21]. Additional challenges include reduced access to primary health care services, resources, and trained health professionals [22]. However, few healthy lifestyle programs have been implemented in rural settings [5, 23], and a systemic review has highlighted that the value of weight gain prevention programs in rural communities has not been established [1]. As such, low-cost, low-intensity weight gain prevention programs are urgently needed in rural settings [20, 21].

Evaluation provides vital insights into how a program achieves efficacy and effectiveness. It also generates essential knowledge to drive implementation and scale-up [24–27] and deliver impact from research investment at the population level [28–32]. Despite this, few evaluations have been applied to weight gain prevention programs, leaving a major knowledge gap [24, 33, 34]. Common barriers to evaluation are lack of funding, time constraints, limited workforce knowledge, skills, and familiarity with evaluation methodologies and the dearth of valid evaluation tools [35].

The RE-AIM evaluation framework (Reach, Effectiveness, Adoption, Implementation, and Maintenance) explores program implementation and generalizability, focusing on the transferability of research findings into clinical practice and policy. The RE-AIM framework was developed specifically to evaluate health promotion interventions and encourages data collection at both the individual and organizational level [36, 37]. The RE-AIM framework has been applied numerous times to health promotion programs and to childhood obesity prevention programs [38, 39], highlighting its value and methodological rigor. However, on assessment of health promotion research programs and grants employing the RE-AIM framework, less than 10% had applied the complete RE-AIM reporting criteria [40, 41]. Most measured one or two RE-AIM dimensions only. The authors of the RE-AIM framework have recently developed criteria to assess the use of the RE-AIM framework [40].

The Healthy Lifestyle Program (HeLP-her) is an evidence-based weight gain prevention program for reproductive-aged women, which here was adapted for rural settings in the HeLP-her Rural trial, with an embedded hybrid implementation and evaluation design [42]. This manuscript aims to (a) provide a summation of evaluation and implementation results from the large-scale HeLP-her Rural randomized controlled trial, utilizing the RE-AIM framework and (b) assess the application of the RE-AIM framework to the HeLP-her Rural program (utilizing the RE-AIM assessment criteria) [40], ultimately aiming to inform implementation and scale-up of obesity prevention programs broadly.

## Methods

### Program design, setting, theory, and implementation

The efficacy of the HeLP-her program has been established previously in two large RCT’s in a community setting for urban-dwelling women and in an antenatal clinic setting for pregnant women [4, 17]. Here, we have used an integrated community cluster RCT design to adapt and implement the HeLP-her program in relatively disadvantaged rural communities in the state of Victoria, Australia (HeLP-her Rural). As previously described, rural town selection was based on population size (2000–10,000 people) and distance from Melbourne central business district (CBD; towns located 100–400 km from CBD). Study randomization occurred at the town (cluster) level and analysis at the individual level. Overall, 41 rural townships met these criteria and were randomized to intervention or control towns. Randomization was conducted by the study biostatistician using a computer-generated randomization list. The primary outcome of HeLP-her Rural was the difference in weight gain between control and intervention groups at 12 months. Study methodology is published elsewhere [42].

#### The HeLP-her Rural program

In summary, the program involved a 1-year active intervention followed by a 1-year observation phase (yet to report). Control participants received a single general group health information session. The intervention aimed to improve participant’s self-management capacity through skill development including goal setting, problem solving, and relapse prevention underpinned by the self-determination theory [43] and motivational interviewing [44]. Participants received the program via mixed delivery modes with minimal personal contact (one group session) and lifestyle advice delivered remotely (phone coaching, text messages, a program manual, and a website) (see Table 1 for further details). The study and embedded implementation-evaluation program were approved by the Monash Health Research Ethics Committee for research involving humans, and all participants provided written informed consent, project No. 12034B. Written consent was provided by all participants.

**Table 1:** HeLP-her Rural implementation and delivery

Community engagement
Regional government departments and community and school leaders were contacted by email, and a follow-up phone call was made. They were invited to support the program implementation by providing introductions to key community groups and assistance with recruitment and providing facilities for program delivery.
Program setting and facilitation
The program was facilitated by three tertiary qualified health professionals with expertise in nutrition, physical activity, and evidence-based practice, and all had worked within the Australian health sector previously. Program facilitators underwent a 1-day training day, led by the program leader, which covered the HeLP-her program theory and practical component, as well as provided motivational interviewing techniques.
Program theory and delivery
The program was designed to be low intensity and focused on participants making small long-term sustainable behavior changes. In this program, 41 rural communities were randomized to intervention or control groups. The control participants attended a single general group health information session. The intervention participants received lifestyle advice through mixed delivery modes including (i) limited personal contact: one group session and (ii) remotely, consisting of one phone coaching session, monthly text message reminders, and a program manual. The delivery methods were designed to reinforce program messages, appeal to various learning styles, and minimize program costs.
Group session
One 60-min group session was held with 8–15 women at community locations such as schools or halls. Facilitators delivered general health information plus simple health messages. Facilitators using an interactive model and supported by the program manual worked through examples of behavioral self-management skills including setting health priorities, problem solving, and self-monitoring, focusing on small changes to behavior.
Program manual
The manual included simple information to improve knowledge and included activities to develop self-management skills such as problem solving, goal setting, and action planning. The participants completed the activities during the interactive group session and were then requested to work through manual activities in their own time.
Phone coaching
Each participant was provided a single 20-min phone coaching session at 16 weeks post intervention commencement. The phone coaching session was delivered by trained coaches to assist completion of manual activities and reinforce program health messages.
SMS text messages and support
One text message was sent every 4 weeks in line with program messages, to remind the participants of the key program messages and goals.

### Program evaluation design and theory

This manuscript reports on the results of the embedded program evaluation using an effectiveness-implementation hybrid study design and involved the simultaneous testing of clinical outcomes and implementation strategies. This research design is thought to facilitate “more rapid translational gains, more effective implementation strategies and promote the collection of useful information for decision makers for scale-up” [45]. This study design is most appropriate for use when effectiveness of the program of interest has been established previously; such is the case for the HeLP-her program. The HeLP-her Rural evaluation aimed to explore program implementation strategies in general within complex systems, assessing implementation rigor. Within this evaluation, process evaluation measures included program fidelity, recruitment strategies, dose of the program delivered and received, program acceptability, and contextual factors influencing program implementation. A summative evaluation investigated overall program effectiveness, quality, community outcomes, and potential for future scale-up. Results on program effectiveness (weight changes), implementation (dose delivered and received, fidelity, recruitment, and program context) and the potential for program sustainability at the organization level have been previously published [46–50]. However, these results are also summarized here to address all elements of the RE-AIM framework.

### Program recruitment and implementation

**Fig. 1:**
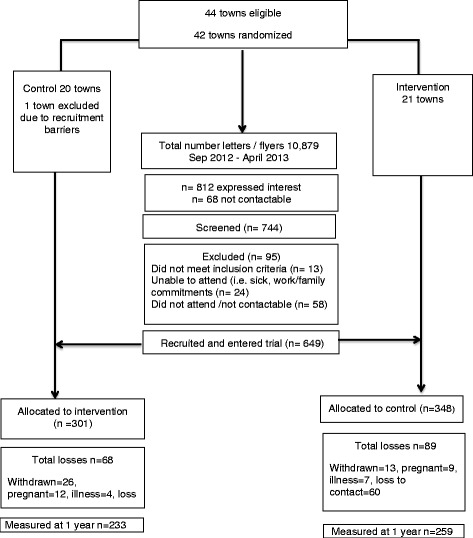
HeLP-her Rural CONSORT Diagram

Recruitment occurred from September 2012–April 2013 (Fig. 1) [49]. Participant recruitment strategies were underpinned by a comprehensive communication and engagement plan and were deliberately simple and low cost to reflect community practice. To assist implementation, we focused on community integration within existing structures and engaged rural communities at multiple levels including local government departments, health services, primary schools, kindergartens, and community groups. Participants were recruited through the distribution of an invitation letter and flyer to women, and research staff visited each township to provide information in person to potential participants. All women aged 18–55 living in the 41 selected communities were invited by letter and community flyers to participate in this program. Program recruitment strategies are described elsewhere [50] (Table 1).

### Data collection methods

The HeLP-her Rural evaluation used mixed method data collection design (Additional file 1) [47]. These included the following: (1) sourcing and analyzing administration data, (2) checklists and log books completed by program leaders, (3) observations by research team, (4) anthropometric data, (5) questionnaires completed by participants, (6a) semi-structured interviews with a sub-group of participants, (6b) semi-structured interviews with rural stakeholders, and (7) an evaluation self-audit.

1. Administrative data and contextual data
  To explore program reach and context, data from the Australian Bureau of Statistics (ABS) measuring Socio-Economic Indexes for Areas (SEIFA) of relative disadvantage was utilized [51]. Potential scores ranged from 1 to 10 with a lower score indicating a greater level of social disadvantage relating to household total income, education attained, and unemployment rates.
2. Program checklists and log books
  Program specific process evaluation checklists were developed in conjunction with the research team field notes to assess implementation fidelity, recruitment, dose delivered, and program context. Program log books were kept to monitor program communication with organizational program partners.
3. Observations by the research team
  The intervention was delivered by a research team (dieticians and exercise physiologists), working in pairs, with one researcher delivering the intervention, the other observing implementation, collecting data, completing checklists and offering feedback to ensure delivery consistency. Program fidelity was addressed by all researchers undergoing a 1-day training workshop (August 2012) delivered by an experienced trainer (CB) and receiving ongoing support and utilizing program developed presentations and resources, promoting consistency of program delivery (Table 1).
4. Anthropometric data
  Weight, height, and waist and hip circumference measurements were collected by the trained team at baseline, 12 months (end of active intervention), and 24 months (end of observation).
5. Questionnaires completed by participants
  All participants completed program devised questionnaires at baseline (prior to intervention commencement) and 12 and 24 months including items on demographic characteristics, socio-cultural and physical environment, health status, and program satisfaction. Participant program satisfaction (overall program, information provided, program delivery, and support provided) was assessed on a program-devised Likert scale (1–5) with higher scores representing greater satisfaction.
6. Semi-structured (qualitative) participant and stakeholder interviews
  In-depth semi-structured interviews were conducted with a sub-group of participant’s 6 months post intervention commencement. A criteria-based, convenience sampling approach as previously described [47, 48] involved women from ten communities (six intervention and four control). One trained researcher conducted all interviews (SK), guided by an interview schedule (Additional file 2). Forty-five participant interviews were conducted until data saturation was met, determined when no new ideas emerged from the interviews, as per standard methods [52].
  Local stakeholders were identified as those who had a direct interest in the development, delivery, and outcomes of prevention programs and included local government employees, clinical health care providers (general practitioners, allied health, community nurses), and non-clinical health providers as previously described [46]. These stakeholders had not directly implemented the HeLP-her Rural program, rather enabled access to local networks for the research team during program implementation. Stakeholders were asked to provide insight into the enablers, barriers, and strategies that would promote program implementation for weight gain prevention. Prior to interviews, each stakeholder was provided with an overview of the HeLP-her study background, the design, staffing, intensity, and implementation plan. Twenty-four semi-structured telephone interviews were conducted led by one researcher (SK) (Additional file 3).
7. Evaluation self-audit (meta-evaluation) of the HeLP-her Rural program
  A meta-evaluation refers to the auditing and assessment of a program evaluation, determining if the evaluation has produced credible and justifiable conclusions [53]. We applied a criteria-based tool to assess the application of the RE-AIM framework to the HeLP-her Rural evaluation, developed by Kessler et al. [40]. This tool outlines a minimum set of “core” items (*n* = 31) producing total and item-specific scores for each of the five RE-AIM domains. This corresponds to a comprehensive application of the framework, termed “fully developed use of RE-AIM”. Items that are not applicable to the program were excluded from the total calculated score (Table 2) [40].

**Table 2:** Meta-evaluation of HeLP-her Rural using criteria developed by RE-AIM

Reach	
A. Participant exclusion criteria (% excluded)	Based on predefined exclusion criteria, less than 12% of the participants (*n* = 95) were excluded post screening (Fig. 1).
B. Percentage who participate	We recruited 649 women into the HeLP-her program or ~10% *n* of the potential target population.
C. Participants characteristics versus nonparticipants	The women involved were representative of the broader Australian regional population (income and education).
D. Qualitative methods	We qualitatively explored program reach (Fig. 2).
Scoring: “Fully Developed Use” 1. (B) and (C) and at least one other item (A or D)	=Fully Developed Use (A + B + C + D): total of (4/4)
Efficacy/effectiveness	
A. Primary outcome measures	At 1-year, the mean weight change in controls was +0.44 kg and in intervention groups was −0.48 kg, a between group difference of −0.92 kg (95% CI −1.67 to −0.16).
B. Measure of broader outcomes	A broad range of outcomes are described elsewhere (food intake, physical activity, self-efficacy, quality of life)
C. Robustness across sub-groups	The intervention showed equally efficacy across various age, BMI, income, and education sub-groups.
D. Attrition (%)	The study retention was 76% at 1 year (Fig. 1).
E. Qualitative methods	Program effectiveness was explored qualitatively.
Scoring: Fully Developed Use 1. Has (A), (B), (C), and (D)	=Fully Developed Use (A + B + C + D + E): total of 5/5 “Yes”
Adoption (setting level)	
A. Setting exclusions (% or reasons)	Yes, one control town was excluded due to difficultly with participant recruitment. This was because recruitment was conducted during peak farming times “harvesting” (Fig. 1).
B. Percentage of settings approached that participated	We contacted 311 local stakeholders and 95% (n = 311) agreed to partner with the HeLP-her program, assisting implementation (Table 3).
C. Characteristics of settings participating versus nonparticipation	Not explored. However, township selection was based on randomization techniques.
D. Use of qualitative methods	Semi-structured stakeholder interviews were conducted.
Scoring: “Fully Developed Use”—adoption setting 1. Must have (B) and (C) and at least one other item (A or D)	=Partially Developed (A + B + D + E): total score of 4/5
Adoption-staff level—not applicable	
Scoring: “Fully Developed Use”—adoption-researchers	N/A
Implementation	
A. Percentage of full delivery or full calls	Comprehensive process evaluation results revealed strong implementation fidelity and high dose delivered.
A. Program adaptions	Implementation was standardized across communities as per study protocol with minor adaptations reported previously.
B. Cost of intervention	Comprehensive economic evaluation is underway.
C. Consistency of researchers, time, and setting	Comprehensive process evaluation indicated implementation consistency.
D. Qualitative methods applied	Program implementation was explored at the community and organizational level with high program acceptability
Scoring: “Fully Developed Use”—implementation: 1. Have (A), (C), and (D) plus at least one more item (B or E)	Fully Developed Use = (A + B + C + D + E): total of 5/5
Maintenance—individual	
A. Primary outcome after final intervention	As above, anthropometric data was collected at baseline and 12 and 24 months with results pending.
B. Measure of broader outcomes, multiple criteria at follow-up	Data analysis collected at 0 and 12 months with food intake, physical activity, self-efficacy and self-management. These outcomes measures will be again explored at 24 months.
C. Robustness data—sub-group effects over the long term	24-month data analysis planned with results pending.
D. Attrition (%)	24-month data analysis planned with results pending.
Scoring: “Fully Developed Use”—maintenance—individual: has (A), (B), (C), and (D)	Fully Developed Use = (A) + (B) + (C) + (D): total of 4/4
Maintenance—setting	
A. Program continuation 6 months post study completion	The HeLP-her program has been endorsed by the Victoria local government preventative health taskforce
B. How program was adapted	N/A
C. Discussion of alignment to organization mission	Exploration undertaken with stakeholders, highlighting that prevention orientated program aligns with local organizational values.
D. Use of qualitative methods.	Stakeholder interviews conducted exploring potential for program continuation and “scale-up”.
Scoring: “fully developed use”—maintenance-setting 1. Has (A) and at least 1 more item (B, C, or D)	Fully Developed Use = (A) + (C) + (D) = 3/3
Entire RE-AIM model scoring	
Reach	Fully Developed Use = (A + B + C + D): total of 4/4 “Yes”
Effectiveness	Fully Developed Use = (A + B + C + D + E): total of 5/5 “Yes”
Adoption	Partial Developed (A + B + D + E): total of 4/5 “Yes”
Implementation	Fully Developed Use = (A + B + C + D + E): total of 5/5 “Yes”
Maintenance: individual	Fully Developed Use = (A) + (B) + (C) + (D): total of 4/4 “Yes”
Maintenance: setting:	Fully Developed Use = (A) + (C) + (D): total of 3/3 “Yes”
Total score: 25/26 = 96% across all RE-AIM dimensions	

### Data analysis

The statistical analysis plan has been previously reported as has statistical methods applied [42, 49]. Data analysis was conducted using STATA and SPSS version 19.0 for Windows. The trial was designed to have a statistical power of 80% to detect a difference of 1.0 kg in weight between groups at 1-year with the use of a two-sided test at a significance level of 0.05. Results are presented as mean (SD) for continuous and relative frequencies for categorical data.

Qualitative transcripts were analyzed thematically, with grounded theory principles of analysis enabling the identification, coding, and categorization of primary data patterns. All transcripts were independently analyzed and coded by two investigators, assisted by the NVivo Software program (QSR International Pty Ltd. Version 10, 2012, Victoria, Melbourne).

## Results

Results are presented in accordance with the RE-AIM dimension and associated key evaluation questions informed by previous literature [33, 37, 40].

### Reach

#### Q1: To what extent did the program reach the target group?

**Fig. 2:**
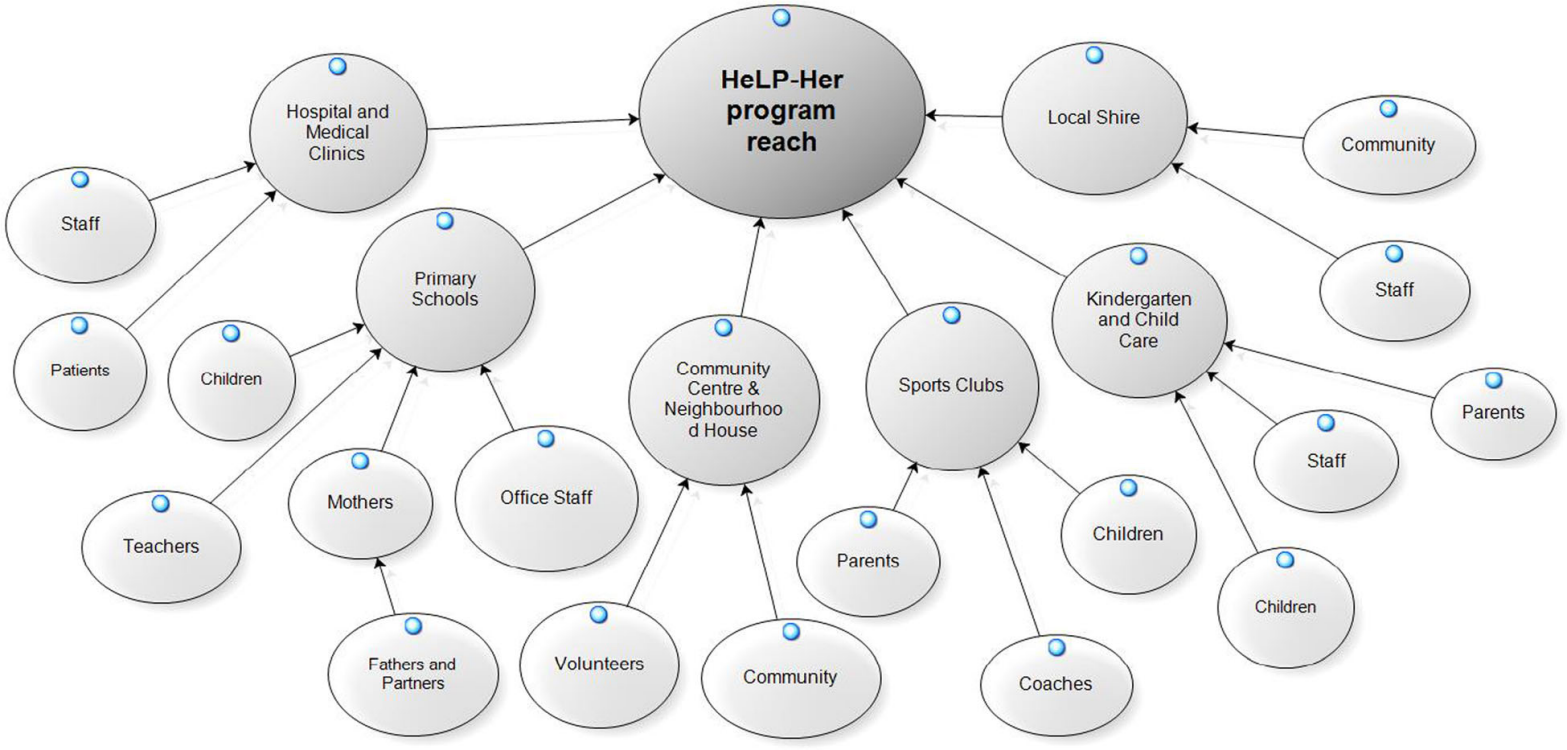
The HeLP-her Rural program community reach. Broad program reach at both the community and organizational levels was achieved by this program. Groups that engaged with the HeLP-her Rural program and their settings included local government agencies, health workers (community health centers, medical clinics, and hospitals), community groups (women’s organizations, neighborhood houses, and sports clubs), education groups (primary schools, kindergartens, and child care centers), and private groups (local businesses and recreational centers)

**Fig. 3:**
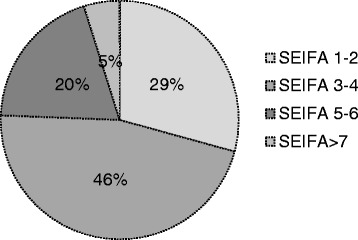
Program reach according to socio-economic disadvantage. This program reached townships of significant socio-economic disadvantage with 75% of townships engaged having a SEIFA index of less than 4 (potential score range of 1–10 with lower scores indicating greater levels of social disadvantage relating to household total income, education attained, and unemployment rates). Overall, 29% of townships reached had a SEIFA index of 1–2, 46% of townships reached had a SEIFA index of 3–4, 20% of townships reached had a SEIFA index of 5–6, and only 5% of townships reached had a SEIFA index of greater than 7

Broad program reach at both the community and organizational level was achieved. Groups that engaged readily included local government agencies, health workers (community health centers, medical clinics, and hospitals), community groups (women’s organizations, neighborhood houses, and sports clubs), education groups (primary schools, kindergartens, and child care centers), and private groups (local businesses and recreational centers) (Fig. 2). As previously reported, *n* = 649 women were recruited, representing ~10% of the eligible target population [47, 50]. Based on predefined and limited exclusion criteria, less than 12% of volunteers (*n* = 95) were excluded post screening (Fig. 1). This program reached townships of significant socio-economic disadvantage with 75% of townships having a SEIFA index of less than 4 (potential score range of 1–10 with lower scores indicating greater disadvantage) (Fig. 3).

The baseline age and BMI of participants was 39.6 ± 6.7 years and 28.8 ± 6.9 kg/m^2^, respectively. The participants had diverse education levels, household sizes, and income levels and were representative of the broader Victorian regional and rural population for women of a similar age (25–54 years) for income and education [50, 54, 55].

#### Q2. What were the key motivators and barriers to program engagement at the participant level (reach)?

As previously described in qualitative analysis, motivators for program participation were convenience of the program location and perceived program utility such as weight management and optimisation of lifestyle choices, as well as attending the program with peers. Barriers to engagement included lack of anonymity, self-consciousness, and segregated social networks in rural settings [50].

#### Q3: Are healthy lifestyles contagious amongst family members and the social networks of program participants?

Qualitative participant interviews highlighted that the HeLP-her Rural program prevention messages reached beyond participants to their families and households. Of the intervention participants interviewed, all reported influencing the health behaviors of their household to various degrees such as increasing water, fruit, and vegetables consumption and physical activity frequency and limiting high-fat snack foods and takeaways.

It’s a roll on effect because if you’re eating healthy yourself and doing healthy meals it follows on to the kids – [intervention participant]

Participants unable to influence their household health choices described barriers which primarily related to unsupportive or resistant family members.

(My husband) he’s my resistance band. He’s like, no, you can have Coke, you can have pies. It’s very hard (for me)- [control participant]

I’ve got two kids that are very fussy eaters…At the end of the day I can’t be bothered with the arguments and whinging - [control participant]

In contrast, women were less likely to influence the health behaviors of their social networks, citing personal and social reasons. Personal reasons related to feelings of “embarrassment,” wanting to maintain “privacy,” and “low confidence”.

My weight is something I’m very embarrassed about so I tend not to discuss it with friends. I don’t suppose my friends are struggling with it as I am - [control participant]

Social barriers to broader program reach included lack of socially acceptability to talk to friends about their weight and lifestyle choices as they do not want to be perceived as “self-absorbed” and “talking about myself all the time.” Other participants described that as many of their friends were already “very active” and “pretty healthy,” there was no need to discuss program learnings. Social isolation was another commonly described reason for not influencing social networks. Others noted beliefs that their friends were “unsupportive” and disinterested, “none of my friends are really into exercise and healthy lifestyles”.

Consistent with our qualitative findings, quantitative questionnaire data collected from intervention (*n* = 230) and control participants (*n* = 207) at 12 months showed that 62% of the participants reported influencing health behaviors of their families. However, there was no statistical significant difference between groups. The participants were most likely to influence their children (38%), friends (34%), and partners (30%).

### Effectiveness

#### Q4. Was the HeLP-her Rural program efficacious at preventing weight gain in intervention participants?

As previously reported, HeLP-her Rural prevented weight gain at 1 year with the mean weight change from baseline in the control group at +0.44 kg (95% CI −0.09 to 0.97) and the intervention group at −0.48 kg (95% CI −0.99 to 0.03), with a between-group difference of −0.92 kg (95% CI −1.67 to −0.16) or −0.88 kg (95% CI −1.62 to −0.13) adjusted for baseline values and clustering [49].

#### Q5. What were the enablers and barriers to behavior change sustainability within this program?

Behavior change continuation was facilitated by participant’s ability to apply the core program messages such as setting achievable behavior change goals, problem solving, and relapse prevention. Improved health knowledge, internal motivation, self-efficacy, and internal accountability all supported continued behavior change as previously described [48].

### Adoption

#### Q7. How many and which organizations supported HeLP-her Rural program implementation?

We partnered with more than 300 local organizations across the 41 rural communities with at least three partners from various community sectors in each community. Our partnering success rate was 95% amongst local stakeholders (Table 3). However, local partners were not required to deliver the intervention, rather to support program implementation and delivery. Local partners were highly valuable and reasons included the following: (1) providing local insights regarding community socio-cultural influences, (2) assisting program recruitment through promotion to professional and social networks, distribution of promotional material, and validation and legitimisation of the program, and (3) assisting program delivery (provision of venues to host the program). As local partners did not directly deliver the HeLP-her program, adoption by staff could not be directly assessed.

**Table 3:** HeLP-her Rural partnership development

Types of organizations	Number of organizations contacted	Number of organizations that supported HeLP-her rural	Success rate of partnerships developed (%)
Local government employees			
●Local government area managers ●Regional mangers ●Community development officers	66	60	91
Primary and Catholic schools			
●School principals ●Vice principals ●Administrative researchers	95	90	95
Kindergartens/child care centers			
●Director ●Administrative researchers	60	58	97
Private businesses			
●Gym owners/personal trainers ●Local workplaces	12	10	91
Sports club			
●Cricket club ●Netball club ●Swimming club	20	20	100
Health centers/primary Care			
●Health service managers ●Health promotion officers ●Clinical researchers (GP’s, nurses, allied health)	38	35	92
Community services			
●Neighborhood houses ●Rural women’s organizations	25	25	100.0
Totals	321	304	94.5

#### Q8. Do stakeholders value weight gain prevention programs?

According to stakeholders, prevention programs were highly valuable and aligned with rural organizational health priorities.

I would encourage my managers to support [prevention programs] because, the demand on the service for weight management is huge and it is particularly women - [Local dietician]

Stakeholders partnered with the HeLP-her Rural program team due to the underlying robust theory, evidence base, and simplistic low-intensity design.

The model (program theory) sounded like something worth promoting. That low commitment program… It sounded like a good program to support – [Health Service Manager].

In addition, many stakeholders valued the use of remote methods to deliver lifestyle advice, “It’s new, innovative ideas and I think that using digital medium is a great way.”

Indeed, due to the low intensity of the program and minimal resources required, many stakeholders discussed that their organization would likely have sufficient capacity to implement such a program.

I think we could probably deliver a program like that ourselves, with the correct information, I mean and tailoring it to what we’ve got. Actually I was sitting in on the program and thinking you know we could do this –[Community nurse]

Stakeholders provided recommendations to improve program delivery and optimize future scale-up of prevention programs. As previously reported, key recommendations included the development of multi-level partnerships, ongoing mentoring relationships via electronic communication, delivery of programs amongst outlying rural townships, and the provision of a suite of implementation resources to support cost and time-efficient implementation [46].

### Implementation

#### Q9. To what extent had the HeLP-her Rural program been implemented as per the study protocol?

Process evaluation revealed strong implementation fidelity and high dose delivered and received, confirmed through administration records, researchers observations, and completed program devised checklists, as previously reported. This highlighted the acceptability of low-intensity healthy lifestyle programs with mixed face-to-face and remote delivery modes in this population group. Qualitative participant interviews revealed that group education sessions were most valued, followed by text messages and phone coaching. Overall, delivery of lifestyle advice through multiple delivery modes (group sessions, phone coaching, text messages, and a program manual) was recommended by participants to optimize program acceptability and accommodate diverse learning styles [47]. A full economic analysis is underway based on a pending 2-year data analysis and will be reported elsewhere.

#### Q10. Were participants satisfied with the HeLP-her Rural program experience?

Qualitative participant interviews reported a high level of program acceptability.

I think [the program] is fantastic and just like the fact that you held it in the first place, I thought oh wow, there’s people that are actually out there to help and yes, it felt like a privilege – [intervention participant]

The 1-year satisfaction surveys showed high participant satisfaction with 82% of the participants agreeing they would recommend this program, with more intervention participants recommending the program (90 versus 73%, *P* < 0.05) and being satisfied with program support than the controls (84 versus 61%, *P* < 0.05).

### Maintenance

#### Q11. Is there evidence of an organizational demand for program continuation post study completion?

The HeLP-her program has been identified as an evidence-based community program by the Victorian State Government as part of a state-wide system-based approach to tackling the rising rates of obesity and preventing obesity-related conditions [56]. This demonstrates the value of a self-management lifestyle program to prevent weight gain and the need for comprehensive evaluation and implementation evidence.

#### Q12. Utilizing a criteria-based meta-evaluation audit tool, does the HeLP-her Rural program measure all “core” RE-AIM dimensions?

Employing Kessler’s et al. RE-AIM meta-evaluation tool, this evaluation assessed all “core” elements of the RE-AIM framework, producing a score of 96% for “fully developed use across all RE-AIM dimensions” (Table 2) [40]. We were unable to assess the domain “adoption at the researcher and staff level” as this was not applicable.

## Discussion

The HeLP-her, low-intensity healthy lifestyle program has now been delivered in a range of settings and populations in a series of RCT’s where it effectively improves lifestyle and prevents weight gain [4, 17, 49]. The HeLP-her Rural program achieved broad program reach across 41 rural townships with most participants reporting influencing some of the health behaviors of their families. Program implementation was supported by 300 multi-level partnerships with partners valuing program theory and low-intensity program design. Stakeholders reported capacity to locally implement and sustain the program. The HeLP-her Rural program prevented a weight gain of nearly 1 kg on average amongst women living in rural Australia. The US Agency for Healthcare Research and Quality defines a weight difference of 0.5 kg between groups as clinically significant and meaningful. Notably, a modeling study estimated that a 1-kg weight loss, if applied across the USA population, could avoid 2 million cases of diabetes, 1.5 million cases of cardiovascular disease, and more than 73,000 cases of cancer [57]. These findings add new information on effective weight gain prevention strategies, mirroring current clinical guidelines and addressing international health priorities to halt the obesity epidemic.

The RE-AIM framework was developed to facilitate translation of research findings into improved population health outcomes, focusing on increasing the reporting of implementation strategies and external program validity [41]. A systematic review of the RE-AIM framework shows broad application to health promotion programs mainly around chronic disease. However, the framework has been inconsistently and sometimes incorrectly applied across all five dimensions [40, 41]. Reporting all RE-AIM dimensions and criteria is critical with interdependent relationships important for public health impact [41]. Updated RE-AIM criteria [40] recommend qualitative research to improve understanding of results, yet this is rarely employed [40, 41]. Here, we address key evaluation and obesity lifestyle prevention gaps by applying qualitative and quantitative research methods and extending evaluation to all RE-AIM dimensions. Subsequently, increasing the generalizability of our results to obesity prevention programs more broadly.

Here, we targeted rural women based on high rates of weight gain, risk of obesity and related complications, adverse impact on maternal and child short- and long-term health, and relative disadvantage for rural women. Despite reported difficulties in engaging socio-economic disadvantaged groups into research [58], we reached women from disadvantaged communities who were representative of regional women of a similar age (25–54 years) based on income, household size, and education [54]. We note that participants reported directly influencing their household’s lifestyle behaviors, such as improving fruit and vegetable intake and physical activity frequency and reducing discretionary food and takeaway consumption. Research exploring the relationship between lifestyle changes in women participating in healthy lifestyle programs and family reach is limited. Further research exploring measurable impact of participant’s reports of influence with members of the household is needed. However, our results support the potential importance of targeting reproductive-aged women [59–61], due to their influential role in influencing household lifestyle behaviors and food choices [62].

The HeLP-her Rural program and evaluation has addressed the dearth of literature surrounding the acceptability of low-intensity weight gain prevention programs in young women [59, 63]. The program has successfully engaged 1127 women across three large RCT’s, emphasizing that low-intensity programs appeal to women from highly diverse socio-economic, ethnic, and educational backgrounds [4, 17, 47]. Furthermore, we have shown that a combination of delivery modes (face-to-face, phone coaching, text messages, and program manuals) optimizes program acceptability and delivers effectiveness. This is in keeping with a recent systematic review reporting the effectiveness of using diverse e-health methods (emailing, texting, phone apps, and websites) to prevent and manage obesity [64]. Supporting the high value of weight gain prevention programs in women, 92% of intervention participants reported that they would recommend this program. Conversely, intensive weight loss programs are generally poorly received with low engagement and high attrition rates [65]. The value and effectiveness of low-intensity weight gain prevention programs demonstrated here can inform the design and execution of prevention programs and strategies more broadly.

Implementing, scaling up, and sustaining evidence-based lifestyle programs in diverse real-world settings are notoriously challenging, yet they are fundamental to improve public health outcomes [66]. Here, we report on the strong potential for program adoption and scale-up, related to broad community reach, effectiveness, low-intensity program design, and high acceptability at the participant and organizational levels. Supporting the value of the prevention-based programs at the organizational level, we successful partnered with local organizations. Stakeholders valued the underlying program theory and design and alignment of the program objectives with their organizational health priorities because this was a unique professional development opportunity. These partnerships were essential to optimizing program implementation, especially in relation to participant engagement. As previously reported [46], stakeholders recommended strategies for optimizing program sustainability and scale-up including building local capacity, developing partnerships and minimizing implementation costs [67, 68]. Moving forward, the HeLP-her program is ready for delivery by local rural health professionals with an interest in weight gain prevention. To date, training of rural communities by the HeLP-her Rural research team has occurred to support professional capacity building of the rural workforce. Funding has been provided by the Australian government preventative health taskforce to refine resources, engage, and adapt considering ethnic diversity and for initial steps in community roll-out [69]. However, to enable program sustainability long-term funding is required, and partnerships with funders in a range of setting are currently in development.

### Study strengths and limitations

Study strengths include utilizing a robust mixed methods evaluation design with an underpinning theoretical framework and a meta-evaluation that revealed all “core” RE-AIM elements were assessed. However, we note that “adoption” at the staff level was unable to be measured within this context due to program design. Limitations include that our program checklists exploring program fidelity and contextual influences were completed by researchers involved in the trial, rather than independent evaluators. Contextual factors and characteristics could also have been further explored. Furthermore, whilst comprehensive 2-year weight data and program economic analysis are underway, these data are needed to inform program maintenance. Given a large amount of data needed to meet all “core” RE-AIM dimensions, a staged approach to data collection and analysis in RE-AIM evaluation is common.

## Conclusions

Evidence-based obesity prevention programs such as the HeLP-her healthy lifestyle program have potential for population level scale-up, with broad reach beyond direct participants, demonstrated effectiveness across a range of populations and settings, low-intensity design, and high acceptability at the community and stakeholder levels. Here, we report strong program implementation rigor and comprehensive application of the RE-AIM framework. Moving forward, there is a clear need to redefine lifestyle program success if we are to deliver public health impact. Success must extend beyond effectiveness to incorporate program reach, adoption and potential for implementation and sustainability in diverse real-world settings. This is critical to inform policy and scale-up of lifestyle programs to address the current obesity epidemic.

## Additional files

Additional file 1: The HeLP-her Rural evaluation applying the RE-AIM framework. (DOCX 24.6 KB)
Additional file 2: Participant semi-structured interview schedule. (DOCX 15.7 KB)
Additional file 3: Stakeholder semi-structured interview schedule. (DOCX 15.2 KB)
