# Supplementary material for: Evaluation of a large healthy lifestyle program: informing program implementation and scale-up in the prevention of obesity
Source: Implement Sci. 2016 Nov 24;11:151. doi: 10.1186/s13012-016-0521-4 (PMC5121947; doi:10.1186/s13012-016-0521-4)
Supplement: Additional file 1: — The HeLP-her Rural evaluation applying the RE-AIM framework. (DOCX 24.6 KB) [file 13012_2016_521_MOESM1_ESM.docx]

**Additional file 1: The HeLP-Her Rural evaluation applying the RE-AIM framework**

| **Key evaluation questions** | **Measures/Indicators** | **Methods of data collection** | 0 mth | 4 mth | 1year | 2years |
| --- | --- | --- | --- | --- | --- | --- |
| **REACH** | | | | | | |
| To what extent was the program received by the target group? | Document percentage of participants recruited based on valid target population denominator.  Characteristics of participants compared to the target population. | Program devised checklists | [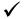](http://www.google.com.au/url?sa=i&source=images&cd=&cad=rja&docid=n4hO7JYM1_yRlM&tbnid=SUz3SKd6AxkbZM:&ved=0CAgQjRwwAA&url=http://www.firekill.co.uk/products/fire_extinguisher.aspx&ei=_OObUsHnFcbDkwW5-oH4Ag&psig=AFQjCNExFqJB2nRA6n3uQw-faqt2fO_wWw&ust=1386034556392219) |  |  |  |
|  |  | Administrative data | [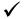](http://www.google.com.au/url?sa=i&source=images&cd=&cad=rja&docid=n4hO7JYM1_yRlM&tbnid=SUz3SKd6AxkbZM:&ved=0CAgQjRwwAA&url=http://www.firekill.co.uk/products/fire_extinguisher.aspx&ei=_OObUsHnFcbDkwW5-oH4Ag&psig=AFQjCNExFqJB2nRA6n3uQw-faqt2fO_wWw&ust=1386034556392219) |  |  |  |
| What participant recruit methods were successful in encouraging program participation?  What were the barriers and enablers to program recruitment? | Documented all resources employed to attract participation (flyers, advertising) stakeholder engagement. | Program devised checklists | [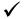](http://www.google.com.au/url?sa=i&source=images&cd=&cad=rja&docid=n4hO7JYM1_yRlM&tbnid=SUz3SKd6AxkbZM:&ved=0CAgQjRwwAA&url=http://www.firekill.co.uk/products/fire_extinguisher.aspx&ei=_OObUsHnFcbDkwW5-oH4Ag&psig=AFQjCNExFqJB2nRA6n3uQw-faqt2fO_wWw&ust=1386034556392219) |  |  |  |
|  | Qualitative exploration of enablers and barriers to program engagement. | Participant interviews |  | [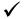](http://www.google.com.au/url?sa=i&source=images&cd=&cad=rja&docid=n4hO7JYM1_yRlM&tbnid=SUz3SKd6AxkbZM:&ved=0CAgQjRwwAA&url=http://www.firekill.co.uk/products/fire_extinguisher.aspx&ei=_OObUsHnFcbDkwW5-oH4Ag&psig=AFQjCNExFqJB2nRA6n3uQw-faqt2fO_wWw&ust=1386034556392219) |  |  |
| Did the HeLP-her Rural program influence the wider community’s health behaviours (family, friends, co-workers of participants)? | Number of women reporting influencing knowledge and/or behaviours of others who did not participate in the program. | Quantitative questionnaires |  |  | [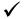](http://www.google.com.au/url?sa=i&source=images&cd=&cad=rja&docid=n4hO7JYM1_yRlM&tbnid=SUz3SKd6AxkbZM:&ved=0CAgQjRwwAA&url=http://www.firekill.co.uk/products/fire_extinguisher.aspx&ei=_OObUsHnFcbDkwW5-oH4Ag&psig=AFQjCNExFqJB2nRA6n3uQw-faqt2fO_wWw&ust=1386034556392219) |  |
|  |  | Participant interviews |  | [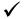](http://www.google.com.au/url?sa=i&source=images&cd=&cad=rja&docid=n4hO7JYM1_yRlM&tbnid=SUz3SKd6AxkbZM:&ved=0CAgQjRwwAA&url=http://www.firekill.co.uk/products/fire_extinguisher.aspx&ei=_OObUsHnFcbDkwW5-oH4Ag&psig=AFQjCNExFqJB2nRA6n3uQw-faqt2fO_wWw&ust=1386034556392219) |  |  |
| **EFFECTIVNESS** | | | | | | |
| Was the HeLP-Her Rural program effective at preventing weight gain? | Primary outcome measurements, comparison of changes in weight in controls versus intervention. | Anthropometric data | [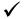](http://www.google.com.au/url?sa=i&source=images&cd=&cad=rja&docid=n4hO7JYM1_yRlM&tbnid=SUz3SKd6AxkbZM:&ved=0CAgQjRwwAA&url=http://www.firekill.co.uk/products/fire_extinguisher.aspx&ei=_OObUsHnFcbDkwW5-oH4Ag&psig=AFQjCNExFqJB2nRA6n3uQw-faqt2fO_wWw&ust=1386034556392219) |  | [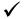](http://www.google.com.au/url?sa=i&source=images&cd=&cad=rja&docid=n4hO7JYM1_yRlM&tbnid=SUz3SKd6AxkbZM:&ved=0CAgQjRwwAA&url=http://www.firekill.co.uk/products/fire_extinguisher.aspx&ei=_OObUsHnFcbDkwW5-oH4Ag&psig=AFQjCNExFqJB2nRA6n3uQw-faqt2fO_wWw&ust=1386034556392219) |  |
| Did intervention participants modify diet and exercise frequency in comparison to controls? | Cancer Council Food Frequency Questionnaire. | Quantitative questionnaires | [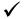](http://www.google.com.au/url?sa=i&source=images&cd=&cad=rja&docid=n4hO7JYM1_yRlM&tbnid=SUz3SKd6AxkbZM:&ved=0CAgQjRwwAA&url=http://www.firekill.co.uk/products/fire_extinguisher.aspx&ei=_OObUsHnFcbDkwW5-oH4Ag&psig=AFQjCNExFqJB2nRA6n3uQw-faqt2fO_wWw&ust=1386034556392219) |  | [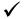](http://www.google.com.au/url?sa=i&source=images&cd=&cad=rja&docid=n4hO7JYM1_yRlM&tbnid=SUz3SKd6AxkbZM:&ved=0CAgQjRwwAA&url=http://www.firekill.co.uk/products/fire_extinguisher.aspx&ei=_OObUsHnFcbDkwW5-oH4Ag&psig=AFQjCNExFqJB2nRA6n3uQw-faqt2fO_wWw&ust=1386034556392219) | [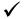](http://www.google.com.au/url?sa=i&source=images&cd=&cad=rja&docid=n4hO7JYM1_yRlM&tbnid=SUz3SKd6AxkbZM:&ved=0CAgQjRwwAA&url=http://www.firekill.co.uk/products/fire_extinguisher.aspx&ei=_OObUsHnFcbDkwW5-oH4Ag&psig=AFQjCNExFqJB2nRA6n3uQw-faqt2fO_wWw&ust=1386034556392219) |
|  | International Physical Activity Questionnaire |  |  |  |  |  |
|  | Participant semi-structured interviews. | Participant interviews |  | [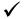](http://www.google.com.au/url?sa=i&source=images&cd=&cad=rja&docid=n4hO7JYM1_yRlM&tbnid=SUz3SKd6AxkbZM:&ved=0CAgQjRwwAA&url=http://www.firekill.co.uk/products/fire_extinguisher.aspx&ei=_OObUsHnFcbDkwW5-oH4Ag&psig=AFQjCNExFqJB2nRA6n3uQw-faqt2fO_wWw&ust=1386034556392219) |  |  |
| What were the enablers and barriers to participant behaviour change? | Qualitative exploration of enablers and barriers to behaviour change. | Participant interviews |  | [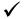](http://www.google.com.au/url?sa=i&source=images&cd=&cad=rja&docid=n4hO7JYM1_yRlM&tbnid=SUz3SKd6AxkbZM:&ved=0CAgQjRwwAA&url=http://www.firekill.co.uk/products/fire_extinguisher.aspx&ei=_OObUsHnFcbDkwW5-oH4Ag&psig=AFQjCNExFqJB2nRA6n3uQw-faqt2fO_wWw&ust=1386034556392219) |  |  |
| **ADOPTION** | | | | | | |
| Do the stakeholders value the HeLP-her program and prevention focused programs? | Exploration of stakeholder interest in program implementation. | Stakeholder interviews |  | [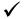](http://www.google.com.au/url?sa=i&source=images&cd=&cad=rja&docid=n4hO7JYM1_yRlM&tbnid=SUz3SKd6AxkbZM:&ved=0CAgQjRwwAA&url=http://www.firekill.co.uk/products/fire_extinguisher.aspx&ei=_OObUsHnFcbDkwW5-oH4Ag&psig=AFQjCNExFqJB2nRA6n3uQw-faqt2fO_wWw&ust=1386034556392219) | [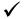](http://www.google.com.au/url?sa=i&source=images&cd=&cad=rja&docid=n4hO7JYM1_yRlM&tbnid=SUz3SKd6AxkbZM:&ved=0CAgQjRwwAA&url=http://www.firekill.co.uk/products/fire_extinguisher.aspx&ei=_OObUsHnFcbDkwW5-oH4Ag&psig=AFQjCNExFqJB2nRA6n3uQw-faqt2fO_wWw&ust=1386034556392219) |  |
| How many and which organizations participated or supported program implementation? To what extent? | Percentage of settings, organizations and individuals who agreed to support or participate in the HeLP-her program. | Administrative data | [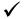](http://www.google.com.au/url?sa=i&source=images&cd=&cad=rja&docid=n4hO7JYM1_yRlM&tbnid=SUz3SKd6AxkbZM:&ved=0CAgQjRwwAA&url=http://www.firekill.co.uk/products/fire_extinguisher.aspx&ei=_OObUsHnFcbDkwW5-oH4Ag&psig=AFQjCNExFqJB2nRA6n3uQw-faqt2fO_wWw&ust=1386034556392219) |  |  |  |
| **IMPLEMENTATION** | | | | | | |
| To what extent was the program implemented as per the study protocol? Were all intended program elements provided to participants? (Fidelity, dose delivered & received) | Evidence that all program elements were delivered and an assessment of program delivery in relation to study protocol.  Adaptions to the intervention during study implementation. Consistency of implementation across settings. | Program devised checklists administration data | [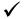](http://www.google.com.au/url?sa=i&source=images&cd=&cad=rja&docid=n4hO7JYM1_yRlM&tbnid=SUz3SKd6AxkbZM:&ved=0CAgQjRwwAA&url=http://www.firekill.co.uk/products/fire_extinguisher.aspx&ei=_OObUsHnFcbDkwW5-oH4Ag&psig=AFQjCNExFqJB2nRA6n3uQw-faqt2fO_wWw&ust=1386034556392219) | [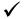](http://www.google.com.au/url?sa=i&source=images&cd=&cad=rja&docid=n4hO7JYM1_yRlM&tbnid=SUz3SKd6AxkbZM:&ved=0CAgQjRwwAA&url=http://www.firekill.co.uk/products/fire_extinguisher.aspx&ei=_OObUsHnFcbDkwW5-oH4Ag&psig=AFQjCNExFqJB2nRA6n3uQw-faqt2fO_wWw&ust=1386034556392219) | [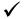](http://www.google.com.au/url?sa=i&source=images&cd=&cad=rja&docid=n4hO7JYM1_yRlM&tbnid=SUz3SKd6AxkbZM:&ved=0CAgQjRwwAA&url=http://www.firekill.co.uk/products/fire_extinguisher.aspx&ei=_OObUsHnFcbDkwW5-oH4Ag&psig=AFQjCNExFqJB2nRA6n3uQw-faqt2fO_wWw&ust=1386034556392219) | [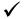](http://www.google.com.au/url?sa=i&source=images&cd=&cad=rja&docid=n4hO7JYM1_yRlM&tbnid=SUz3SKd6AxkbZM:&ved=0CAgQjRwwAA&url=http://www.firekill.co.uk/products/fire_extinguisher.aspx&ei=_OObUsHnFcbDkwW5-oH4Ag&psig=AFQjCNExFqJB2nRA6n3uQw-faqt2fO_wWw&ust=1386034556392219) |
|  |  | Participant interviews |  | [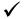](http://www.google.com.au/url?sa=i&source=images&cd=&cad=rja&docid=n4hO7JYM1_yRlM&tbnid=SUz3SKd6AxkbZM:&ved=0CAgQjRwwAA&url=http://www.firekill.co.uk/products/fire_extinguisher.aspx&ei=_OObUsHnFcbDkwW5-oH4Ag&psig=AFQjCNExFqJB2nRA6n3uQw-faqt2fO_wWw&ust=1386034556392219) |  |  |
|  |  | Researchers observations | 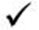 | [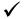](http://www.google.com.au/url?sa=i&source=images&cd=&cad=rja&docid=n4hO7JYM1_yRlM&tbnid=SUz3SKd6AxkbZM:&ved=0CAgQjRwwAA&url=http://www.firekill.co.uk/products/fire_extinguisher.aspx&ei=_OObUsHnFcbDkwW5-oH4Ag&psig=AFQjCNExFqJB2nRA6n3uQw-faqt2fO_wWw&ust=1386034556392219) | [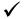](http://www.google.com.au/url?sa=i&source=images&cd=&cad=rja&docid=n4hO7JYM1_yRlM&tbnid=SUz3SKd6AxkbZM:&ved=0CAgQjRwwAA&url=http://www.firekill.co.uk/products/fire_extinguisher.aspx&ei=_OObUsHnFcbDkwW5-oH4Ag&psig=AFQjCNExFqJB2nRA6n3uQw-faqt2fO_wWw&ust=1386034556392219) | [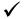](http://www.google.com.au/url?sa=i&source=images&cd=&cad=rja&docid=n4hO7JYM1_yRlM&tbnid=SUz3SKd6AxkbZM:&ved=0CAgQjRwwAA&url=http://www.firekill.co.uk/products/fire_extinguisher.aspx&ei=_OObUsHnFcbDkwW5-oH4Ag&psig=AFQjCNExFqJB2nRA6n3uQw-faqt2fO_wWw&ust=1386034556392219) |
| Were participants satisfied with HeLP-her program (information, level of support)? | Exploration of participant program satisfaction. | Participant interviews |  | [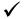](http://www.google.com.au/url?sa=i&source=images&cd=&cad=rja&docid=n4hO7JYM1_yRlM&tbnid=SUz3SKd6AxkbZM:&ved=0CAgQjRwwAA&url=http://www.firekill.co.uk/products/fire_extinguisher.aspx&ei=_OObUsHnFcbDkwW5-oH4Ag&psig=AFQjCNExFqJB2nRA6n3uQw-faqt2fO_wWw&ust=1386034556392219) |  |  |
|  |  | Quantitative questionnaires |  |  | [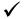](http://www.google.com.au/url?sa=i&source=images&cd=&cad=rja&docid=n4hO7JYM1_yRlM&tbnid=SUz3SKd6AxkbZM:&ved=0CAgQjRwwAA&url=http://www.firekill.co.uk/products/fire_extinguisher.aspx&ei=_OObUsHnFcbDkwW5-oH4Ag&psig=AFQjCNExFqJB2nRA6n3uQw-faqt2fO_wWw&ust=1386034556392219) | [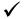](http://www.google.com.au/url?sa=i&source=images&cd=&cad=rja&docid=n4hO7JYM1_yRlM&tbnid=SUz3SKd6AxkbZM:&ved=0CAgQjRwwAA&url=http://www.firekill.co.uk/products/fire_extinguisher.aspx&ei=_OObUsHnFcbDkwW5-oH4Ag&psig=AFQjCNExFqJB2nRA6n3uQw-faqt2fO_wWw&ust=1386034556392219) |
| **MAINTENANCE** | | | | | | |
| Was the HeLP-her Rural effective at preventing weight gain at 24 months? | Changes in participants measured weight at 24 months (differences between control and intervention participants). Measurements of program attrition. | Anthropometric data | [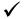](http://www.google.com.au/url?sa=i&source=images&cd=&cad=rja&docid=n4hO7JYM1_yRlM&tbnid=SUz3SKd6AxkbZM:&ved=0CAgQjRwwAA&url=http://www.firekill.co.uk/products/fire_extinguisher.aspx&ei=_OObUsHnFcbDkwW5-oH4Ag&psig=AFQjCNExFqJB2nRA6n3uQw-faqt2fO_wWw&ust=1386034556392219) |  | [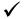](http://www.google.com.au/url?sa=i&source=images&cd=&cad=rja&docid=n4hO7JYM1_yRlM&tbnid=SUz3SKd6AxkbZM:&ved=0CAgQjRwwAA&url=http://www.firekill.co.uk/products/fire_extinguisher.aspx&ei=_OObUsHnFcbDkwW5-oH4Ag&psig=AFQjCNExFqJB2nRA6n3uQw-faqt2fO_wWw&ust=1386034556392219) | [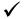](http://www.google.com.au/url?sa=i&source=images&cd=&cad=rja&docid=n4hO7JYM1_yRlM&tbnid=SUz3SKd6AxkbZM:&ved=0CAgQjRwwAA&url=http://www.firekill.co.uk/products/fire_extinguisher.aspx&ei=_OObUsHnFcbDkwW5-oH4Ag&psig=AFQjCNExFqJB2nRA6n3uQw-faqt2fO_wWw&ust=1386034556392219) |
| What demand exists for continuation of program? | Exploration of potential for program sustainability and scale-up (enablers, barriers and recommendations).  Alignment between organizations missions and HeLP-her | Stakeholder interviews |  | [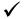](http://www.google.com.au/url?sa=i&source=images&cd=&cad=rja&docid=n4hO7JYM1_yRlM&tbnid=SUz3SKd6AxkbZM:&ved=0CAgQjRwwAA&url=http://www.firekill.co.uk/products/fire_extinguisher.aspx&ei=_OObUsHnFcbDkwW5-oH4Ag&psig=AFQjCNExFqJB2nRA6n3uQw-faqt2fO_wWw&ust=1386034556392219) | [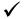](http://www.google.com.au/url?sa=i&source=images&cd=&cad=rja&docid=n4hO7JYM1_yRlM&tbnid=SUz3SKd6AxkbZM:&ved=0CAgQjRwwAA&url=http://www.firekill.co.uk/products/fire_extinguisher.aspx&ei=_OObUsHnFcbDkwW5-oH4Ag&psig=AFQjCNExFqJB2nRA6n3uQw-faqt2fO_wWw&ust=1386034556392219) |  |
