# Supplementary material for: Evaluation of a large healthy lifestyle program: informing program implementation and scale-up in the prevention of obesity
Source: Implement Sci. 2016 Nov 24;11:151. doi: 10.1186/s13012-016-0521-4 (PMC5121947; doi:10.1186/s13012-016-0521-4)
Supplement: Additional file 2: — Participant semi-structured interview schedule. (DOCX 15.7 KB) [file 13012_2016_521_MOESM2_ESM.docx]

| **Interview schedule** |
| --- |
| 1. Motivators, enablers and barriers for participant recruitment (Reach)  - Participants motivators for attendance - Reported potential local barriers to participation and strategies to address barriers - Program expectations |
| 1. Impact of the HeLP-her program on the broader community  - Exploration of whether the program messages, information and/or resources had influenced the health behaviours of participants families and social networks - Barriers to disseminating program messages to the wider community |
| 1. Program effectiveness at the participant level  - Perceived effectiveness of the program demonstrated through knowledge gains, behaviour changes and shifts in attitudes - Barriers to program effectiveness i.e. lack of personal motivation, confidence in the program, personal factors |
| 1. Exploration of program engagement and utilisation  - Utilisation of the various program components (group session, phone coaching, SMS text messages and manual) - Preferred method of receiving health information |
| 1. Program satisfaction and acceptability (Implementation)  - Design of the program - Facilitator (Urban or locally based) - Location and timing of the program education sessions - Information provided - Level of support provided |

**Additional file 2: Participant semi-structured interview schedule**
