# Supplementary material for: Evaluation of a large healthy lifestyle program: informing program implementation and scale-up in the prevention of obesity
Source: Implement Sci. 2016 Nov 24;11:151. doi: 10.1186/s13012-016-0521-4 (PMC5121947; doi:10.1186/s13012-016-0521-4)
Supplement: Additional file 3: — Stakeholder semi-structured interview schedule. (DOCX 15.2 KB) [file 13012_2016_521_MOESM3_ESM.docx]

**Additional file 3: Stakeholder semi-structured interview schedule**

| **Overview of stakeholder’s roles within the community and community profiling**   1. Can you describe your role in your community? 2. Can you briefly describe the community you work in? 3. From your experiences would you consider the community to be socially connected and engaged? 4. Which factors influenced your choice to assist with implementation our evidence based weight gain prevention program? |
| --- |
| **Service availability**   1. What organised programs or services exist in your community that promote healthy eating, exercise and are there any obesity prevention and management programs available? 2. What health programs are missing from your community? What other healthy lifestyle programs would you like to see available in your community? |
| **Stakeholder’s value and interest in running prevention programs and potential for continuation**   1. Does a preventive healthy lifestyle program fit in with your organisations current health plan/priority area or strategy? 2. Do you think women in your community value preventive healthy lifestyle programs? |
| **Enablers and barriers to healthy lifestyle program implementation**   1. Can you describe any present enablers of obesity prevention programs implementation in your community? 2. What would the major barriers be to implementing obesity prevention programs in your community? |
| **Recommended strategies to optimise healthy lifestyle program implementation**   1. Can you describe and recommend ways that we could support or improve the ease of implementation an obesity prevention programs in your community? Prompts: training, resources, support, program etc. |
